# High seroprevalence of Rift Valley fever phlebovirus in domestic ruminants and African Buffaloes in Mozambique shows need for intensified surveillance

**DOI:** 10.1080/20008686.2017.1416248

**Published:** 2017-12-17

**Authors:** Belisário Moiane, Lourenço Mapaco, Peter Thompson, Mikael Berg, Ann Albihn, José Fafetine

**Affiliations:** ^a^ Department of Para-clinical, Veterinary Faculty, Eduardo Mondlane University, Maputo, Mozambique; ^b^ Department of Biomedical Sciences and Veterinary Public Health, Swedish University of Agricultural Sciences, Uppsala, Sweden; ^c^ Section for Environment and Biosecurity, National Veterinary Institute, Uppsala, Sweden; ^d^ Directorate of Animal Sciences, Institute of Agrarian Research, Maputo, Mozambique; ^e^ Department of Production Animal Studies, Faculty of Veterinary Science, University of Pretoria, Pretoria, Republic of South Africa

**Keywords:** Rift Valley fever phlebovirus, seroprevalence, Mozambique, domestic ruminants, African buffaloes

## Abstract

**Introduction**: Rift Valley fever (RVF) is an arthropod-borne disease that affects both animals and humans. RVF phlebovirus (RVFPV) is widespread in Africa and Arabian Peninsula. In Mozambique, outbreaks were reported in South; seroprevalence studies performed in livestock and water buffaloes were limited to central and south regions. We evaluated the seroprevalence of RVFPV among domestic ruminants and African buffaloes from 7 of 10 provinces of Mozambique, to understand the distribution of RVFPV and provide data for further RVF control programs.

**Materials and methods**: A total of 1581 blood samples were collected in cattle, 1117 in goats, 85 in sheep and 69 in African buffaloes, between 2013 and 2014, and the obtained sera were analyzed by ELISA.

**Results and discussion**: The overall seroprevalence of RVFPV domestic ruminants and African buffaloes was 25.6%. The highest was observed in cattle (37.3%) and African buffaloes (30.4%), which were higher than in previous studies within Mozambique. In south and central regions, the overall seroprevalences were higher (14.9%–62.4%) than in the north.

**Conclusion**: This study showed the presence of anti-RVFPV antibodies in animals from all sampled provinces, suggesting that RVFPV is actively circulating among domestic ruminants and African buffaloes in Mozambique, therefore surveillance should be intensified.

## Introduction

Rift Valley fever (RVF) is an endemic disease on the African continent, where it has a negative impact on the livestock production, due to high mortality rate among infected animals, trade bans of animals from affected areas, and its zoonotic impact [1]. RVF is caused by a tri-segmented RNA virus of negative polarity, belonging to *Phenuiviridae* family, genus *Phlebovirus* [2]. RVF phlebovirus (RVFPV) [2] is transmitted by arthropods, mainly mosquitoes of the genera *Aedes* and *Culex* [3,4] to many species such as sheep, goats, cattle, camels, horses [5], wildlife species [6] and humans [7,8].

RVFPV was first isolated in Kenya, during an outbreak that occurred in sheep in 1931 [9]. Since then, RVFPV has spread to many African countries [10,11], Saudi Arabia [7,12] and Yemen outside Africa [11].

RVF is a transboundary disease, and recent outbreaks were reported in Niger and Uganda [2016, 11,13], Mauritania (2010, 2012–2015, 14,15,16), Senegal (2013/14) and South Africa and Namibia (2008–2011, 17,18,19,20), Kenya, Tanzania and Somalia in 2006 [21,22]. High number of deaths among humans and livestock were reported during these outbreaks [11]

Animals infected with RVFPV may remain asymptomatic or experience fever, diarrhea, nasal discharge, weakness, abortion, decrease in milk production and death. Susceptibility to infection is mostly age and species-related. Young animals are more susceptible to infection when compared to adults [23].

In humans, RVFPV was reported to cause a self-limiting febrile illness, but also more severe symptoms as encephalitis, retinopathy and deaths [24,25]. RVFPV was also found to be linked with miscarriage in 54% (15/28) of women in a cross-sectional study performed at the Governmental Hospital of Port Sudan between June 2011 and November 2012, following a sudden onset of abortions [26].

In Mozambique the first outbreak of RVF occurred in 1969 in the Provinces of Gaza and Maputo, where a great number of cattle and goats died and a large number of abortions were observed due to virus infection [27]. A small outbreak of RVF has been reported in Goba (Namaacha district), Maputo Province, where 56% (49/88) cattle were positive for RVFPV IgG and 24% for IgM [28]. Furthermore, RVFPV RNA was detected in 12 serum samples by RT-PCR. In the same study 2/26 and 2/13 sera collected in Xai-Xai and Chibuto districts, respectively, in Gaza Province, were RVFPV RNA positive [28], suggesting that this virus was actively circulating among domestic ruminants in those areas.

Since 1969 [27], Mozambique has never reported large outbreaks of RVF, however seroepidemiological studies performed in Maputo and Gaza Provinces (South) and Zambezia Province (Central), between 1996 and 2013, found high seroprevalences of RVFPV among domestic ruminants and water buffaloes *(Bubalus* bubalis), which ranged from 21% to 53% [29–33], except for the survey performed in 2010 in sheep (9%) and goats (12%) in Zambezia [34].

The first reported evidence of RVFPV circulation among humans in Mozambique was in 1989, when 2% (28/1163) sera collected from pregnant women in 8 of 10 provinces were positive to RVFPV antibodies [35]. A recent seroepidemiological study performed among acute febrile patients in Southern Mozambique, revealed a higher prevalence of 5% (10/200) RVFPV IgG antibodies, during the heavy rains in 2013 [36]. These reports, in combination with reported animal seroprevalences show that RVF may be an important yet neglected zoonosis in Mozambique. Silent circulation occurs and outbreaks, with severe public health and economic impact in both livestock production sector and human health, may occur during the hot rainy season.

To date, the overall prevalence of RVF in animals in Mozambique is not known. Studies done so far have concentrated on the Provinces of Maputo, Gaza and Zambezia.

In the present study, we evaluated the seroprevalence of RVF throughout Mozambique to understand the distribution of RVFPV among domestic ruminants and African buffaloes from 2013 to 2014, and thus support the veterinary authorities and general public with baseline information on RVF prevalence, which may allow the appropriate decision making for the implementation of preventive programs countrywide.

## Materials and methods

### Study area

**Figure 1.**
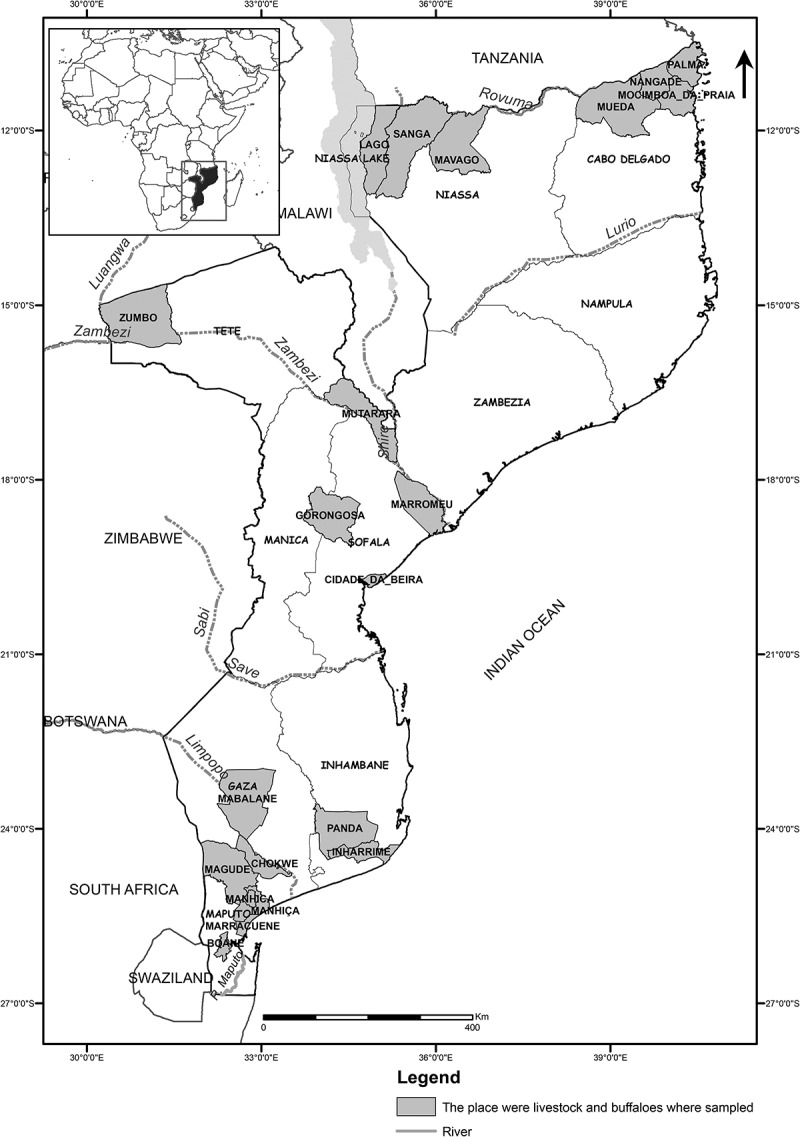
Map of Mozambique showing the study area in each province.

A cross-sectional study was performed on farms located in 7 of 10 Provinces of Mozambique, grouped in three geographic regions, respectively, north (Cabo Delgado and Niassa), central (Sofala and Tete) and south (Inhambane, Gaza and Maputo) (Figure 1). Of the 7 provinces, Gaza and Maputo have reported RVF outbreaks among domestic ruminants [27,28].

Mozambique’s average annual temperatures are around 20°C, the highest, 38–40°C, are recorded between December and February. These temperatures, coinciding with the hot rainy season (October to March), favor high rainfall (between 800–1000 mm in the south, and even higher in the central and northern regions) [37], and thus breeding of RVFPV mosquito vectors [38].

The Country´s largest plains and the largest hydrographic basins occur in the central and southern regions (mainly in the south), namely the Maputo, Incomati, Limpopo and Save rivers in the South, as well as Pungue, Buzi and the great Zambezi Basin in central region, except for the river Lurio and Rovuma that are in the north (Figure 1). During the rainy season these rivers overflow, flooding the plains [37], favoring the breeding of RVFPV mosquito vectors [38–40].

In the north, cattle and goats were sampled in Cabo Delgado Province (Mocimboa da Praia, Mueda, Nangade districts), and only goats were sampled in Niassa (Lago, Mavago and Sanga districts) (Figure 1).

In central Mozambique, African buffaloes (*Syncerus caffer*) and cattle were sampled in both provinces of Tete (Mutarara and Zumbo districts) and Sofala (Beira, Gorongosa and Marromeu districts), whereas goats were only sampled in Tete for this region (Table 1). Buffalo samples were collected in the Tchuma-tchato special conservation zone in Zumbo district (Tete), Gorongosa National Park in Gorongosa and Marromeu Special Reserve of Buffaloes in Marromeu districts, respectively in (Sofala) (Figure 1).

**Table 1.** Rift Valley fever seroprevalence in Mozambique, 2013–2014, by province and species.

	Province	Cattle		Goats		Sheep		Buffalo		TOTAL	
Region		*n*	Prevalence [95% CI]	*n*	Prevalence [95% CI]	*n*	Prevalence [95% CI]	*n*	Prevalence [95% CI]	*N*	Prevalence [95% CI]
	Cabo Delgado	31	25.8 ^bc^ *	281	**2.5 ^c^ †**					312	**4.8 ^d^**
			[11.9 – 44.6]		[1.0 – 5.1]						[2.7 – 7.8]
North	Niassa			234	**1.3 ^c^**					234	**1.3 ^e^**
					[0.3 – 3.7]						[0.3 – 3.7]
	Tete	30	63.3 ^a^ *	129	**3.9 ^bc^ †**			22	**13.6 ^b^ †**	181	**14.9 ^c^**
			[43.9 – 80.1]		[1.3 – 8.8]				[2.9 – 34.9]		[10.1 – 21.0]
Central	Sofala	543	64.5 ^a^ *					47	**38.3 ^a^ †**	590	**62.4 ^a^**
			[60.3 – 68.5]						[24.5 – 53.6]		[58.3 – 66.3]
	Inhambane			241	**17.8 ^a^**					241	**17.8 ^c^**
					[13.2 – 23.3]						[13.2 – 23.3]
South	Gaza	749	19.2 ^c^ *	223	20.2 ^a^ *	72	18.1 ^a^ *			1,044	**19.3 ^c^**
			[16.5 – 22.2]		[15.1 – 26.1]		[10.0 – 28.9]				[17.0 – 21.9]
	Maputo	228	29.8 ^b^ *	9	22.2 ^ab^ *	13	23.1 ^a^ *			250	**29.2 ^b^**
			[24.0 – 36.2]		[2.8 – 60.0]		[5.0 – 53.8]				[23.6 – 35.3]
	**TOTAL**	1,581	37.3 *	1,117	**9.4 ‡**	85	**18.8 †**	69	30.4 *†	2,852	**25.6**
			[34.9 – 39.7]		[7.8 – 11.3]		[11.2 – 28.8]		[19.9 – 42.7]		[24.0 – 27.3]

**^abcde^** Within columns, seroprevalences with no superscripts in common differ significantly (*p* < 0.05).

*†‡ Within rows, seroprevalences with no symbols in common differ significantly (*p* < 0.05).

The Tchuma-tchato special area, in the Zumbo district of Tete, is located in a medium to high plateau area, upstream Zambezi Valley (200–1000 m above mean sea level), has an annual average temperature of 24–26°C and precipitation between 1200–1400 mm [41].

In Sofala, the Marromeu Reserve is located in the Zambezi Delta plain, with more than 79 rivers and streams of permanent water, average precipitation of 910 mm, temperature 21°C (16–32°C), with a large occurrence of dambos [42], and Gorongosa National Park in the Gorongosa district, with many rivers and streams of permanent water; altitude varying from 300–600 m, above the sea level, and annual average temperature of 22.9°C (16.1–32°C) [43].

In south region, all the three species of domestic ruminants were included in the sampling, in Gaza (Chóckwè, Mabalane and Bilene districts) and Maputo (Boane, Manhiça and Marracuene), except for Inhambane province (Inharrime and Panda), where only goats were sampled (Table 1).

The study was performed under ethical permit number 555/DNSV/2008, issued by the Board of Ethics of the Directorate of the National Veterinary Service, Ministry of Agriculture and Food Security of Mozambique.

### Sampling

A total of 1581 blood samples was collected in cattle, 1117 in goats, 85 in sheep and 69 in African buffaloes (Table 1); the sampling was performed between April 2013 and June 2014.

Cattle, goats and sheep (males and females), both young and adults of mixed breeds (local, exotic and cross-breeds), were gathered and restrained in communal pens located near acaricidal dip tanks, used for the massive chemical control of tick in livestock of both smallholders and commercial farmers in each sampling area. Cattle were considered to be young when age was estimated at <2 years and the cut-off for sheep and goats was <12 months.

African buffaloes, both young (< 2.5 years) and adults (≥ 2.5 years) were chemically immobilized by aerial darting, for subsequent sampling [44].

Animals were bled through venipuncture of the caudal vein, blood samples were collected into vacutainer tubes and thereafter kept on dry ice and transported to the local laboratories, where they were centrifuged to obtain sera. Thereafter, sera samples were stored at −20^º^C, until they were shipped to the Central Veterinary Laboratory in Maputo, followed by storage at the above-mentioned temperature, until further analysis.

According to the Directorate of the National Veterinary Services, as far as possible the sampling locations were selected based on the presence of the risk factors that favor the breeding of RVFPV mosquito vectors (large plains, wetlands, high precipitation rates during the rainy season, a high likelihood for flooding), and risk factors that favor the spread of RVFPV as high livestock density. This indicates that the survey was not random, but was targeted at the likely high prevalence areas. Therefore it may overestimate the general seroprevalence in the country.

### Serological testing

ID Screen® RVF competition multi-species ELISA kit (IDvet, France) that simultaneously detects both RVFPV IgG and IgM was used to test the sera. Briefly, sera samples were added to 96 well plates, coated with a recombinant RVF nucleoprotein (NP). After washing the plates an anti-NP peroxidase conjugate was added to fix the remaining free NP epitopes. Substrate and stop solutions were added, microplates were read at 450 nm, and cut-off values were calculated as recommended by manufacturer.

### Statistical analysis

Seroprevalences were calculated by province and species with 95% confidence interval (CI) and were compared using Fisher’s exact test. Multivariable analysis using logistic regression, aimed at evaluating the association between potential risk factors (location, species, sex and age) and RVF seropositivity among cattle, sheep goats and the African buffaloes was performed. Odds ratio (OR) were calculated and goodness-of-fit of the model was evaluated using the Hosmer-Lemeshow goodness-of-fit test [45].

## Results

The overall seroprevalence was 25.6% (731/2852; 95% CI: 24.0 – 27.3%) and within the regions the central with 51.2% (395/771) had the highest overall prevalence of RVFPV antibodies, followed by south with 21% (323/1535); the lowest was observed in the north, 3.3% (18/546). In the north region the seroprevalence of RVFPV was highest in Cabo Delgado with 4.8% (15/312). Sofala had 62.4% (368/590) and is the province with highest overall seroprevalence for the central region, while the highest RVFPV seroprevalence 29.2% (73/250) for the south region was found in Maputo (Table 1). Across all regions, significant differences in overall seroprevalences of RVFPV were observed between Sofala, Maputo, Cabo Delgado and Niassa (Table1), and between these four provinces and Gaza, Inhambane and Tete. Furthermore, the last three provinces did not differ significantly.

The overall prevalence of anti-RVFPV antibodies was higher for cattle 37.3% (590/1581) and African buffaloes 30.4% (21/69), than in other species and the highest was observed in the central province of Sofala 64.5% (350/543) for cattle and 38.3% (18/47) for African buffaloes. Goats, with 20.2% (45/223) in south province of Gaza had higher prevalence of anti-RVFPV antibodies than those observed in the Tete, Cabo Delgado and Niassa; meanwhile, no differences were observed between Maputo and Tete, as well as between Tete and the north provinces for this species (Table 1).

Sheep were only sampled in Gaza and Maputo provinces, and no significant differences were observed on seroprevalence of RVFPV for this species between the two provinces (Table 1).

African buffaloes´ samples were only available from central region and the seroprevalence of anti-RVFPV antibodies for this species from both Marromeu district (Marromeu National Reserve) and Gorongosa district (Gorongosa National Park) in Sofala province was 38.3% (18/47), higher than the observed in Zumbo district (Tchuma-tchato Special Conservation Zone), in Tete province (Table 1).

In this study, a significant difference was observed regarding the likelihood of the animals being seropositive to RVF, in the provinces of Inhambane, Maputo, Sofala and Tete in relation to Gaza (Table 2). The odds of cattle being seropositive in Sofala and Maputo was, respectively, two and four times higher than the cattle in Gaza province, whereas the odds of seropositivity were lower in Inhambane and Tete (Table 2).

**Table 2.** Factors associated with Rift Valley fever seropositivity in livestock in central and southern Mozambique, 2013–2014.

Variable & level	Odds ratio	95% CI	*p*-value
**Province (region)**			
Gaza (South)	1*	–	–
Inhambane (South)	0.41	0.25 – 0.68	<0.001
Maputo (South)	1.69	1.05 – 2.72	0.032
Sofala (Central)	4.10	3.01 – 5.59	<0.001
Tete (Central)	0.09	0.03 – 0.30	<0.001
**Species**			
Cattle	1*	–	–
Goats	1.13	0.71 – 1.79	0.617
Sheep	0.49	0.25 – 0.95	0.034
**Sex**			
Male vs. female	1.40	0.93 – 2.10	0.106
**Age**			
Adult vs. young	8.94	5.42 – 14.77	<0.001

* Reference level. Hosmer-Lemeshow goodness-of-fit χ^2^_5 df_ = 3.64, *P* = 0.602.

Goats and cattle had the same likelihood of being seropositive (OR = 1.13, *p* = 0.617), however, sheep (OR = 0.49, *p *= 0.034) had lower odds of being seropositive than cattle (Table 2).

There was no significant difference between male and female animals in the odds of being seropositive; however, in relation to age, adult animals were nine times more likely to have been exposed to RVFPV than the younger ones (OR = 8.92, *p* < 0.0001) (Table 2).

## Discussion

This was the first time that the animals in the north of Mozambique (Cabo Delgado and Niassa provinces) have been surveyed for detection of RVF antibodies, and to our knowledge no reports of RVFPV circulation from this region have been available. The decision to include the north region of Mozambique was motivated by recent findings of high RVFPV seroprevalences in Central Zambézia province [34], and the Republic of Tanzania [46], respectively, which are in the neighborhoods, and therefore raised concerns with regards to the RVFPV seroprevalence in livestock within this region.

In this study, the overall seroprevalences of RVFPV for each species (except for goats) were high (Table 1). A similar result was found in a serological survey of anti-RVFPV antibodies in Tanzania where 25.8% (n = 1435) of domestic ruminants (sheep, cattle and goats) were seropositive [47]. The seroprevalence found in this study was higher than that found in cattle and goats in the Union of Comoros (17.5%; n = 191), between 2010–2011 [48].

The high prevalence of antibodies to RVFPV in the central and south Mozambique as compared to the north, may be related to climatic factors, water availability and landscape, which likely influence the amplification and spread of RVFPV in the east and southern African countries [40,49].

The low seroprevalence of RVFPV in the north region can be explained by the predominant high altitude (above 1000 m), dense and closed forest, and low average annual temperatures (<18°C) compared to other regions of the country. Despite the high precipitation (between 800 – 2000 mm) in the hot and rainy season (from October to March) [37], the low temperature may not favor the breeding of the potential vectors of the RVFPV. Another factor may be related to the low density of animals and large dispersion among herds, which may hamper the ability of the vectors to spread the virus [50]. The high seroprevalence observed in Cabo Delgado, for this region, may be explained by the fact that in this province the altitude and forest density decrease eastward (Figure 1), and the annual average temperatures tend to increase (>24°C), which, in comparison to Niassa, may increase the survival rate of RVFPV mosquitos’ vectors, and hence increase the vectorial transmission of RVFPV to the animals.

The lower overall seroprevalence in Tete province as compared to Sofala province, may be related to its relatively hot and dry climate, with lower precipitation rates (below 800 mm, much lower in Zumbo and Mutarara districts) [37], factors that may contribute to poor breeding or lower survival rates of potential RVFPV vectors. However, in some instances, these areas may experience a dramatic increase in the rainfall, in association with El Niño southern oscillation phenomenon, leading to floods and a surge in mosquito population and thus increase the risk of RVFPV transmission to both animals and humans [51].

Unlike Tete, in Sofala province, where the main plain of the central Mozambique occurs, with a great availability of permanent natural sources of water, a humid tropical climate, high likelihood for flooding [37] and favorable temperatures to the proliferation of RVFPV vectors, in addition to the fact that there is high density of both livestock and buffaloes per herd, the risk of infection and vectorial viral spread among animals is high [40]. These factors may explain the observed high RVFPV seroprevalence.

A situation similar to that of Sofala can be observed in relation to the provinces of south Mozambique, which have the largest plain lands in the country [37] with a very high potential for the breeding of RVFPV vectors, especially during the rainy season in which frequent floods occur almost yearly.

On the other hand, intensive agricultural activity is concentrated in these regions, mainly focused on the production of sugarcane and rice in Maputo, Gaza and Sofala. This situation has led to the opening of more irrigation ditches [37], which may have contributed to the increase in mosquito breeding and greater exposure of animals [50].

In the present study, cattle had a higher prevalence of antibodies when compared to goats and sheep (Table 1). The seroprevelance observed for cattle in this studies were higher than those observed in Madagascar 28% (n = 894) in 2009 [52] and 14% (n = 1353) between 2010–2011 [53], Botswana 5.7% (n = 863), in 2010–2011 [54], Rwanda 16.8% (n = 595) between December 2012 and March 2013 [55] and Tanzania 27.7% (n = 756) in 2013 [47]. Studies carried out in the central (Zambezia and Manica) and south (Maputo and Gaza) Mozambique between 1996 and 2013, have shown high seroprevalence of RVF antibodies in cattle (7% – 51.6%), goats (11.6% – 25.1%), and sheep 9.2% – 44.2%) [29–34]. The high density of cattle in the herds in relation to goats and sheep may be one of the contributing factors to their greater susceptibility to RVFPV infection [56]. However, it would be important to evaluate the effect of density of animal species on susceptibility to RVFPV infection in Mozambique. A follow up study on RVF outbreak in South Africa, has found a higher seroprevalence in cattle than goats and sheep raised on the same farm, and this was related to a possible mosquito vector preference to feed on cattle´s blood [57]. Furthermore, the preference of the RVFPV mosquito vectors on cattle than goats and sheep was observed in Kenya [58].

In the present study, a high prevalence was observed in cattle raised in the surroundings of the Tchuma Tchato special conservation zone (in the District of Zumbo in Tete), Marromeu National Reserve and Gorongosa National Park, in Sofala Province (Figure 1), which could indicate that there is a circulation of the RVFPV between domestic ruminants and African buffaloes in Mozambique. However, the importance of wildlife as a potential reservoir for RVFPV has not been determined.

A study performed in northern Botswana, found a prevalence of antibodies to RVFPV in buffaloes (12.7%, n = 150) higher than in cattle (5.7%, n = 863) [54]. In the present study, we did not observe a significant difference between these two species regarding overall seroprevalence (Table 1). However, the seroprevalence of cattle (63.3%, n = 30) was higher than that of buffaloes (12.6%, n = 22) in the province of Tete (Table 1). On the other hand, the overall prevalence in both species was high in our study, when compared to the study done by Jori *et al*. [54].

A high seroprevalence in buffaloes has been demonstrated by many researchers mainly in sub-Saharan Africa especially in South Africa [59,60] and Kenya [61]. In our study, seroprevalence was also high. African buffaloes are known to be a common host of RVFPV [60]. Other study performed in African buffaloes reported lower seroprevalence, 7.5% (34/541) in Zimbabwe [6], compared to this study. Differences in seroprevalence between wildlife populations are likely related to differences in environmental risk factors.

The difference in seroprevalence between buffaloes in Sofala (Marromeu Reserve and Gorongosa National Park) and those in Tete (Tchuma-tchato Special Conservation Area) may be associated with climatic events and factors related to the landscape that influence the type, distribution and the density of potential RVFPV mosquito vectors species [49,62].

The distribution, density and potential of RVFPV transmission by the different potential vectors in different ecological zones is not yet known in Mozambique, but is currently being investigated in southern region.

The overall prevalence of anti-RVFPV antibodies in goats and sheep observed in this study was lower compared to that observed in Tanzania, 22% (117/531) for goats and 30% (44/148) for sheep, respectively [47].

The high seroprevalences of RVFPV in cattle and sheep observed in south Mozambique were also reported for these species in the central Province of Zambezia by Fafetine *et al*. between 2010–2013[34] and Blomström *et al*. in 2013 [33].

In 2013 and 2014 no outbreaks of RVF or clinical cases were reported in livestock and wildlife from all provinces, except for the southern provinces of Gaza and Maputo, where small outbreaks of RVF in the districts Xai-Xai and Chibuto (Gaza) and Goba (Maputo), were reported in 2014. RVFPV was detected in 16 serum samples and 6 tissues from sheep and goat, using quantitative real-time reverse transcription PCR, by amplifying the 490 nucleotide region of the medium segment of RVFPV RNA [28].

Silent circulation, due to subclinical infections with RVFPV, was reported in goats and sheep in the Province of Zambezia [34], cattle and African buffaloes in other regions of sub-Saharan Africa [38]. As a result of RVFPV circulation pattern in Mozambique, small outbreaks and sporadic cases of clinical and or economic importance may be overlooked. Also RVFPV may possibly be a significant cause of human illness.

The location of the province where the animals are raised, the animal species and the age, were identified in this study as being the main risk factors for RVFPV infection between 2013 and 2014.

Older animals were more likely to be infected, suggesting enzootic circulation of RVFPV in Mozambique. Abdalla *et al*. [63], when they evaluated the risk factors for RVFPV infection in Camels (*Camelus dromedaries*) in Sudan, and Blomström *et al*. [33], when investigated the seroprevalence of RVFPV in sheep and goats in Zambezia Province, obtained a similar result. This is commonly seen in infectious diseases and for RVF it may be due to the longer exposure time of the adult animals in the herds, where they are in contact with potential mosquitoes vectors during several rain seasons, with increased risk of acquiring RVFPV infection through bites from infected mosquitoes [33,63]. Very little is known of the ecology of the mosquito vectors of RVFPV in Mozambique.

Whether RVF in the north Mozambique has resulted from the movement of RVFPV infected livestock or wildlife, from previous outbreak areas in both central and south regions, or it originated from different sources cannot be ruled out. Therefore, RVFPV genotyping and phylogeographic analysis could be done in order to address this question.

## Conclusion

The results from this study show the presence of anti-RVFPV antibodies in all sampled provinces, suggesting that RVFPV is actively circulating among domestic ruminants and African buffaloes in Mozambique.

Domestic ruminants in the central and south, as well as African buffaloes from the central regions, respectively, showed a higher prevalence of anti-RVFPV antibodies than those in the north.

Animal age, species and the location were the main factors associated with the odds of exposure to RVFPV in Mozambique during 2013 and 2014.

The results obtained in this study are complementary to those from other reports, and may be useful as baseline information on RVF seroprevalence in both domestic ruminants and African buffaloes in Mozambique. It is clear now that RVF surveillance program in Mozambique must be intensified.
